# Experimental Infection of Cattle with Highly Pathogenic Avian Influenza Virus (H5N1)

**DOI:** 10.3201/eid1407.071468

**Published:** 2008-07

**Authors:** Donata Kalthoff, Bernd Hoffmann, Timm Harder, Markus Durban, Martin Beer

**Affiliations:** * Friedrich-Loeffler-Institut, Greifswald-Insel Riems, Germany

**Keywords:** Highly pathogenic avian influenza, H5N1, cattle, mammals, dispatch

## Abstract

Four calves were experimentally inoculated with highly pathogenic avian influenza virus A/cat/Germany/R606/2006 (H5N1) isolated from a cat in 2006. All calves remained healthy, but several animals shed low amounts of virus, detected by inoculation of nasal swab fluid into embryonated chicken eggs and onto MDCK cells. All calves seroconverted.

Since 1997, an epidemic of highly pathogenic avian influenza virus (HPAIV) subtype H5N1 has spread in Asia, causing fatal infections in poultry, wild birds, and mammals, including humans (*1*). Knowing the susceptibility to HPAIV (H5N1) of mammalian species living in close proximity to humans and poultry, such as members of the family *Bovidae* (e.g., cattle or water buffalo), would be helpful to those developing surveillance plans or determining risk areas. Serologic examinations have indicated that calves might be susceptible to influenza A virus (*2*); however, so far only 1 strain (A/calf/Duschanbe/55/71 H3N2) has been identified as a cattle strain (*3*). A correlation between influenza A virus infection, reduced milk yield, and respiratory symptoms in dairy cows was assumed in the late 1990s (*4*,*5*) and has received recent attention (*6*,*7*). Nevertheless, to our knowledge, no data about the susceptibility of cattle to infections with HPAIV have been reported. Therefore, we experimentally infected bovine calves with HPAIV (H5N1) and collected data about clinical symptoms, viral excretion, and serologic reactions.

## The Study

In 2007, 6 Holstein-Friesian calves, 3 month of age, were obtained from a breeder near Greifswald-Insel Riems, Germany. Their influenza A virus–free status was confirmed, and no avian influenza virus–specific genomes or antibodies were detected. All experiments were performed in the high-containment animal facility (Biosafety Level 3+) at the Friedrich-Loeffler-Institut (trial approval no. LVLM-V/TSD/7221.3-1.1-003/07).

Four of the calves were intranasally inoculated with HPAIV (H5N1) strain A/cat/Germany/R606/2006, which had been isolated from a cat in 2006 (*8*,*9*). The virus was aerosolized in 5 mL cell culture medium containing 10^8.5^ 50% egg infectious dose/mL (third egg passage). The other 2 calves (contacts) were not inoculated but were housed in the same containment room for the duration of the experiment. For 7 days all calves were monitored by physical examination, and pharyngeal swabs were collected and examined for virus excretion.

All 6 calves remained healthy; no specific clinical signs were observed. All nasal swabs were tested with real-time reverse transcription–PCR specific for subtype H5N1 (*10*), and the genomic load was semiquantified by using threshold cycle values. Infectious virus was detected by inoculation of swab fluid into embryonated chicken eggs with 1 subsequent passage and onto MDCK cells (collection of cell lines in veterinary medicine, Friedrich-Loeffler-Institut, Isle of Riems, RIE83).

Nasal swabs from all inoculated calves collected at 1 day postinoculation (dpi) were positive for viral RNA, and 3 of 4 calves shed infectious virus, detected by virus isolation in embryonated chicken eggs (Table 1). Isolation of infectious virus in cell culture was successful in 2 of 4 samples. Furthermore, of 4 inoculated calves, 2 were positive for HPAIV (H5N1) genome copies at 2 dpi, and 1 shed infectious virus. From 3 dpi through 7 dpi, samples from nasal swabs of all animals were negative for viral genome, and all nasal swabs of the 2 contact calves remained negative for HPAIV (H5N1) RNA during the experiment (Table 1).

**Table 1:** Detection of highly pathogenic avian influenza virus (H5N1) in nasal swabs from calves, Germany, 2007*

Calf	Day postinoculation										
	0		1				2				3–7
	PCR†		PCR	Egg	Cell		PCR	Egg	Cell		PCR
Inoculated											
A1	>40		20–30	–	–		30–40	–	–		>40
A2	>40		30–40	+	+		30–40	+	–		>40
A3	>40		20–30	+	+		>40	ND	ND		>40
A4	>40		30–40	+	–		>40	ND	ND		>40
Contact											
K1	>40		>40	ND	ND		>40	ND	ND		>40
K2	>40		>40	ND	ND		>40	ND	ND		>40

*Inoculated calves received highly pathogenic influenza virus (H5N1) strain A/cat/Germany/R606/2006 (*8*,*9*); contact calves were not inoculated but were housed with the inoculated calves. Egg, embryonated chicken eggs; cell, MDCK cell culture; +, virus detected; –, virus not detected; ND, not done. †H5-specific real-time reverse transcription­–PCR; results are shown as range of detected threshold cycle values.

Assuming that susceptible animals should mount an antibody response, we looked for antibodies against the highly conserved and immunogenic nucleoprotein (NP) of type A influenza viruses (*11*). Heat-inactivated (30 min at 56°C) serum samples collected at 0, 7, 14, 21, 28, and 91 dpi were tested for NP-specific antibodies with a licensed commercial ELISA (avian influenza A–blocking ELISA, Pourquier, Montpellier, France) according to the manufacturer’s instructions. Serum samples from 50 HPAIV (H5N1)–negative, nonrelated cattle were used as controls and to confirm the specificity of the ELISA (data not shown).

To quantify the serologic response, we performed virus neutralization (VN) and hemagglutination inhibition (HI) tests with homotypic virus. The VN test was modified according to a previously described procedure (*12*). In brief, bovine serum samples were heat inactivated for 30 min at 56°C, and 3-fold serial dilutions were performed in a 50-μL volume of cell culture medium in 96-well plates. The diluted serum samples were mixed with an equal volume of media containing homotypic influenza virus at a concentration of 10^2^ 50% tissue culture infectious dose/well. After 1 h incubation at 37°C in a 5% CO_2_ humidified atmosphere, 100 μL of Vero cells (African green monkey kidney, collection of cell lines in veterinary medicine, Friedrich-Loeffler-Institut, Isle of Riems, RIE228) at 1.5 × 10^5^/mL was added to each well. The plates were incubated for 3 days at 37°C and 5% CO_2_. Viral replication was assessed by visually scoring the cytopathic effect without staining. Each assay was validated by comparison with positive and negative control serum from chickens and cattle as well as back titration of the used virus dilutions. Previous treatment with periodate (*13*) had no influence on the neutralizing titers. Serum samples were also tested for H5-specific antibodies by HI test with 4 hemagglutinating units of homotypic virus as antigen according to standardized methods (*14*). All serum samples were treated with periodate and heat inactivated to eliminate serum inhibitors. The HI tests were performed with a starting dilution of 1:8 by using a 1% suspension of chicken erythrocytes in a 0.85% saline solution.

The commercial NP-specific ELISA detected influenza A virus–specific antibodies at 14 dpi in 2 of the 4 inoculated animals (Table 2). All 4 animals had positive scores for neutralizing antibodies against the homologous virus at 14 dpi; specific titers ranged from 16 to 51. Furthermore, the HI test detected titers of 8 at 28 dpi in the 4 inoculated calves. At 21 dpi, VN testing indicated that 1 of the contact calves was positive for subtype H5N1–specific antibodies, NP-ELISA results for this calf were questionable, and HI testing did not detect any hemagglutinating antibodies. The other contact calf remained serologically negative throughout the experiment. Finally, 3 months after inoculation, VN test and ELISA clearly indicated seroconversion by all inoculated calves and 1 of the contact calves. In contrast, HI results were negative for all but 2 inoculated animals (Table 2).

**Table 2:** Serologic testing results for highly pathogenic avian influenza virus (H5N1) in calves, Germany, 2007*

Calf	Day postinoculation																						
	0				7				14				21				28				91		
	NP†	VN‡	HI§		NP	VN	HI		NP	VN	HI		NP	VN	HI		NP	VN	HI		NP	VN	HI
Inoculated																							
A1	98	2.3	<3		90	3.3	<3		42	**4**	<3		**26**	**4**	<3		**24**	**6.2**	**3**		**20**	**7**	<3
A2	114	0.4	<3		117	3	<3		69	**5**	<3		**27**	**4**	<3		**22**	**6.2**	**3**		**18**	**6.7**	**3**
A3	118	<1	<3		90	2.7	<3		**28**	**5.7**	<3		**29**	**5**	<3		**21**	**7**	**3**		**15**	**7**	**3**
A4	102	0.7	<3		122	2	<3		**25**	**5.3**	<3		**24**	**5**	<3		**24**	**5.8**	**3**		**19**	**6.3**	<3
Contact																							
K1	96	1.3	<3		88	<1	<3		73	2	<3		44	**4**	<3		40	**4.5**	<3		45	**5**	<3
K2	120	0.7	<3		98	2.3	<3		89	<1	<3		68	<1	<3		76	0.7	<3		50	<1	<3

*Inoculated calves received highly pathogenic influenza virus (H5N1) strain A/cat/Germany/R606/2006 (*8*,*9*); contact calves were not inoculated but were housed with the inoculated calves. Positive results are in **boldface**. †NP, avian influenza A– blocking ELISA against nucleoprotein (Pourquier, Montpellier, France) inhibition % (<35, positive; >45, negative; 35–45, questionable). ‡VN, virus neutralization test (ND100 log_2_); values >4 are considered positive. §HI, hemagglutination inhibition (log_2_); values >3 are considered positive.

## Conclusions

Our findings show that HPAIV (H5N1) has the potential to infect bovine calves, at least after high-titer intranasal inoculation, and that conventional HI tests may underestimate such infections. Furthermore, asymptomatic shedding of HPAIV (H5N1) by infected calves and subsequent seroconversion seem to be possible, and even low levels of HPAIV (H5N1) might be sufficient to induce a detectable antibody response in contact calves. However, the possibility that the infectivity detected in the contact calf at 1 dpi was the result of residual inoculum cannot be ruled out. Although the question whether calf-to-calf transmission of HPAIV (H5N1) occurs could not be definitely answered by our study, bird-to-calf transmission resulting in seroconversion is probable.

The incidence of clinical infections of cattle with HPAIV (H5N1) in disease-endemic regions should be low. However, our data indicate that serum from bovine species would be a valuable source of additional information about transmission events, especially in regions like Asia and Egypt, where HPAIV (H5N1) is endemic and probability of contact between poultry and cattle is high. The NP-ELISA is currently the assay of choice for the evaluation of bovine serum, and the VN test should be used for confirmation.
